# Positive airway pressure therapy and cardiovascular events in obstructive sleep apnoea: an observational clinical cohort study

**DOI:** 10.1016/j.sleep.2025.108732

**Published:** 2025-12-23

**Authors:** Diego R. Mazzotti, Aiyu Chen, Jaejin An, Joanie Chung, Jessica Arguelles, Brendan T. Keenan, Greg Maislin, Bruno Saconi, Alexa J. Watach, Henry Glick, Samuel T. Kuna, Allan I. Pack, Amy M. Sawyer, Dennis Hwang, Jiaxiao Shi

**Affiliations:** a Division of Medical Informatics, Department of Internal Medicine, University of Kansas Medical Center, Kansas City, KS, USA; b Division of Pulmonary Critical Care and Sleep Medicine, Department of Internal Medicine, University of Kansas Medical Center, Kansas City, KS, USA; c Kaiser Permanente Southern California, Department of Research & Evaluation, Pasadena, CA, USA; d Kaiser Permanente Bernard J. Tyson School of Medicine, Pasadena, CA, USA; e Division of Sleep Medicine, Department of Medicine, University of Pennsylvania Perelman School of Medicine, Philadelphia, PA, USA; f School of Nursing, Department of Biobehavioral Health Sciences, University of Pennsylvania, Philadelphia, PA, USA; g Department of Population Health Sciences, Geisinger, Danville, PA, USA; h Division of General Internal Medicine, Department of Medicine, University of Pennsylvania Perelman School of Medicine, USA; i Medicine Department, Corporal Michael J. Crescenz Veterans Affairs Medical Center, Philadelphia, PA, USA

**Keywords:** Obstructive sleep apnoea, Continuous positive airway pressure, Cardiovascular disease, Electronic health records, Treatment adherence

## Abstract

**Aims:**

Studies support the short-term benefit of continuous positive airway pressure (CPAP) therapy on cardiometabolic risk in adults with obstructive sleep apnoea (OSA). Evidence is limited on the benefits of CPAP for preventing acute major adverse cardiovascular events (MACE). This study aimed to assess the association between CPAP use and incidence of MACE in a longitudinal clinical cohort of adults with OSA at a large U.S. healthcare system.

**Methods:**

Adults with OSA (apnoea-hypopnoea index [AHI]≥5) were identified from Kaiser Permanente Southern California between 2018 and 2020 (N = 34,782). MACE was defined as first occurrence of myocardial infarction, stroke, unstable angina, heart failure or cardiovascular death, using validated electronic health record algorithms. CPAP use (h/night) was based on daily telemonitoring data. Inverse probability of treatment weighted Cox proportional hazards models stratified by OSA severity (mild [5≤AHI<15]), moderate-severe [AHI ≥15]) were used to assess associations between CPAP use and MACE.

**Results:**

Among individuals with moderate-severe OSA, those using CPAP <4 h/night (HR [95 % CI] = 0.53 [0.35–0.82]; p = 0.004) or ≥4 h/night (HR [95 % CI] = 0.46 [0.27–0.77]; p = 0.004) had lower MACE incidence compared to those not using CPAP. Increased CPAP use (in hours) was associated with lower MACE incidence in moderate-severe OSA (HR [95 % CI] = 0.90 [0.82–0.98]; p = 0.021). In individuals with mild OSA, CPAP use was not associated with lower MACE incidence.

**Conclusion:**

CPAP use was associated with lower MACE incidence in adults with moderate-severe OSA. Treatment of moderate-severe OSA may have a positive impact on prevention of MACE.

## Graphical Abstract

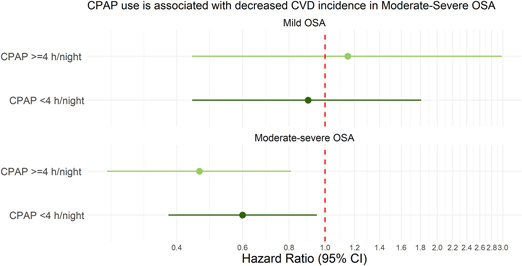

## 1. Introduction

Cardiovascular diseases are prevalent causes of morbidity and remain the leading cause of death in the U.S. [1]. Despite improvements in cardiovascular mortality rates with advanced therapies for established disease, there is an increasing focus on modifying cardiovascular risk factors for primary and secondary prevention [2]. Recently, the relationship between cardiovascular health and sleep, including screening for and treating sleep disordered breathing, has been highlighted [1,3]. Several studies suggest that short sleep duration is associated with incident obesity [4], type 2 diabetes [5], and cardiovascular morbidity [6]. Obstructive sleep apnoea (OSA), a highly prevalent sleep disorder affecting approximately 1 billion people worldwide [7], contributes to these associations. OSA is a systemic disorder [8] and an established risk factor for insulin resistance [9] and cardiovascular diseases [10,11]. A recent meta-analysis found a dose-dependent association between OSA severity and all-cause and cardiovascular mortality [10]. Epidemiological evidence suggests that moderate-severe OSA causes hypertension [12,13] and is associated with the incidence of stroke [14], vascular aging [15], coronary plaque burden [16], myocardial infarction [17], and cardiovascular mortality [11]. Thus, the management of OSA is a potential opportunity to improve cardiovascular health.

Continuous positive airway pressure (CPAP) is the first line treatment for moderate-severe or symptomatic OSA [18]. Randomised controlled trials (RCTs) have established that CPAP improves daytime symptoms, mood, and quality of life in patients with OSA [19,20]. CPAP has also been shown to reduce systolic and diastolic blood pressure [12], especially in patients with resistant hypertension [13], and to have a positive impact on cardiovascular and metabolic functions [11]. Despite the consistent aforementioned evidence of associations between OSA and major adverse cardiovascular events, recent RCTs [21–23] investigating the effect of CPAP on fatal and non-fatal cardiovascular events found that this therapy does not prevent these major adverse cardiovascular outcomes [24]. However, patients enrolled in such RCTs are not representative of the adult population seeking care at sleep centers [25,26] and, therefore, cardiovascular consequences of OSA may play a smaller role. Thus, the effectiveness of CPAP therapy for preventing major adverse cardiovascular events (MACE) in a representative clinical sample of OSA patients has not been robustly evaluated.

Availability of CPAP telemonitoring allows accurate description of patterns of CPAP use and may help inform treatment responses that are more generalisable to the clinical population observed in sleep centers [27]. Obtaining daily CPAP adherence data linked to electronic health records (EHRs) offers opportunities to understand the role of OSA treatment to prevent MACE in a real-world setting. Towards this end, this study assessed the effect of CPAP therapy on the risk of MACE in a longitudinal clinical cohort of adults with OSA within a large healthcare system in Southern California. We hypothesized that adults with OSA using CPAP were less likely to experience MACE than those not using CPAP.

## 2. Methods

### 2.1. Participants

This is an observational retrospective analysis of electronic health record (EHR) and administrative data from Kaiser Permanente Southern California (KPSC), a large, integrated healthcare delivery system serving nearly 5 million members across diverse populations and broad geographic regions in Southern California (United States). KPSC operates as a capitated system with its own network of hospitals, outpatient medical offices, and pharmacies, and all medical and utilisation records are captured in a single comprehensive EHR (Epic Systems). KPSC Sleep Medicine comprises a network of twelve sleep disorder centers that delivers standard of care sleep disorder management that includes diagnostic polysomnography and home sleep apnea testing; CPAP therapy is recommended as first-line therapy for patients with a diagnosis of OSA and emphasized for those with moderate-severe OSA. Adults (age ≥18 years) that underwent diagnostic testing between 01/2018–02/2020 with a diagnostic apnoea-hypopnoea index (AHI4%)≥5 events/hours were included in the study. The index date was the date of the diagnostic sleep study. Eligible participants had at least one year of insurance coverage at KPSC prior to the sleep study date (index date) and did not have a cardiovascular event (see Cardiovascular outcomes below) in the year prior to the index date. Participants were followed until the outcome of interest, disenrollment (gaps >90 days in coverage), death, or study end date (August 2020), whichever occurred first. This study was reviewed by the KPSC Institutional Review Board and determined to be minimal risk; informed consent requirement was waived. Additional details are presented in the Supplemental Material. This study meets all five of the CODE-EHR minimum framework standards for the use of structured healthcare data in clinical research [28].

### 2.2. OSA diagnosis and severity

Polysomnography or home-based sleep apnoea tests were used to identify patients with OSA. AHI was defined as the number of respiratory events (apnoeas ≥10s and hypopnoeas ≥10s associated with ≥4 % oxygen desaturation) per hour of sleep [29]. OSA was defined when the AHI was ≥5 events/hour. OSA severity categories were determined based on the AHI as mild OSA (5≤AHI<15 events/hour) and moderate-severe OSA (AHI≥15 events/hour).

### 2.3. CPAP utilisation

CPAP utilisation data were obtained via an established telemonitoring framework provided by major CPAP vendors. Daily utilisation (i.e., hours of use/night) was averaged across the exposure period to determine three exposure groups: no CPAP (average utilisation of zero or not available), CPAP use <4 h/night, and CPAP use ≥4 h/night. Secondary analysis used average daily hours of CPAP use as an exposure variable with and without quadradic terms (e.g., *CPAP hours* * *CPAP hours*).

### 2.4. Cardiovascular outcomes

Our primary MACE endpoint was a composite of the first occurrence of myocardial infarction (MI), stroke, unstable angina, heart failure or cardiovascular death. We used previously validated algorithms to identify these events (see Supplemental Material).

### 2.5. Covariates

Relevant demographic and clinical covariates were obtained at index or one year prior to the index date (baseline), including: age, sex, race/ethnicity, marital status, neighborhood educational level, neighborhood median household income, body mass index (BMI, in kg/m^2^), Charlson Comorbidity Index (CCI), use of anti-hypertensives and lipid-lowering medications (see Supplemental Table 1), systolic blood pressure, total cholesterol, high-density lipoprotein (HDL) cholesterol, smoking status, and physical activity status based on exercise vital sign [30]. To consider the possibility of healthy user and healthy adherer bias [31], we included a variable representing baseline influenza vaccination rates and a variable measuring “no-show” rates to medical appointments.

### 2.6. Statistical analyses

Baseline demographic and clinical characteristics were described among exposure groups using counts and percentages for categorical data and mean and standard deviation (SD) or median and interquartile range for continuous data. Demographic and clinical characteristic comparions among CPAP utilisation groups were performed using chi-squared tests, Fisher’s exact tests or Kruskal-Wallis’ test. We assessed the association between CPAP utilisation and incidence of MACE, separately among those with mild OSA and moderate-severe OSA. Kaplan-Meier survival curves were used to show MACE-free survival over time. The log-rank test were used to compare survival curves among different CPAP utilisation groups. Covariate-adjusted Cox proportional hazards models were used to evaluate associations between CPAP use and MACE. Further analyses to explore causal inference of the effect of CPAP use on MACE using inverse probability of treatment weighting were also performed. Propensity score (PS) on the likelihood of any CPAP use (<4h or ≥4h/night) were estimated using logistic regression with study covariates for mild and moderate-severe OSA separately. Standardised mean differences were used to check the balance of each characteristics between the two groups, and differences <0.1 were considered as good balance. Inverse probability of treatment weights (1 for the CPAP users and PS/(1-PS) for the non-users) were used together with the CPAP exposure groups in weighted Cox proportional hazards regression models. Results are reported as hazard ratios (HR) and 95 % confidence intervals, representing estimates of the average treatment effect on treated. Results with p < 0.05 were considered statistically significant. Analyses were done using SAS (v.9.4; SAS Institute).

## 3. Results

**Fig. 1.**
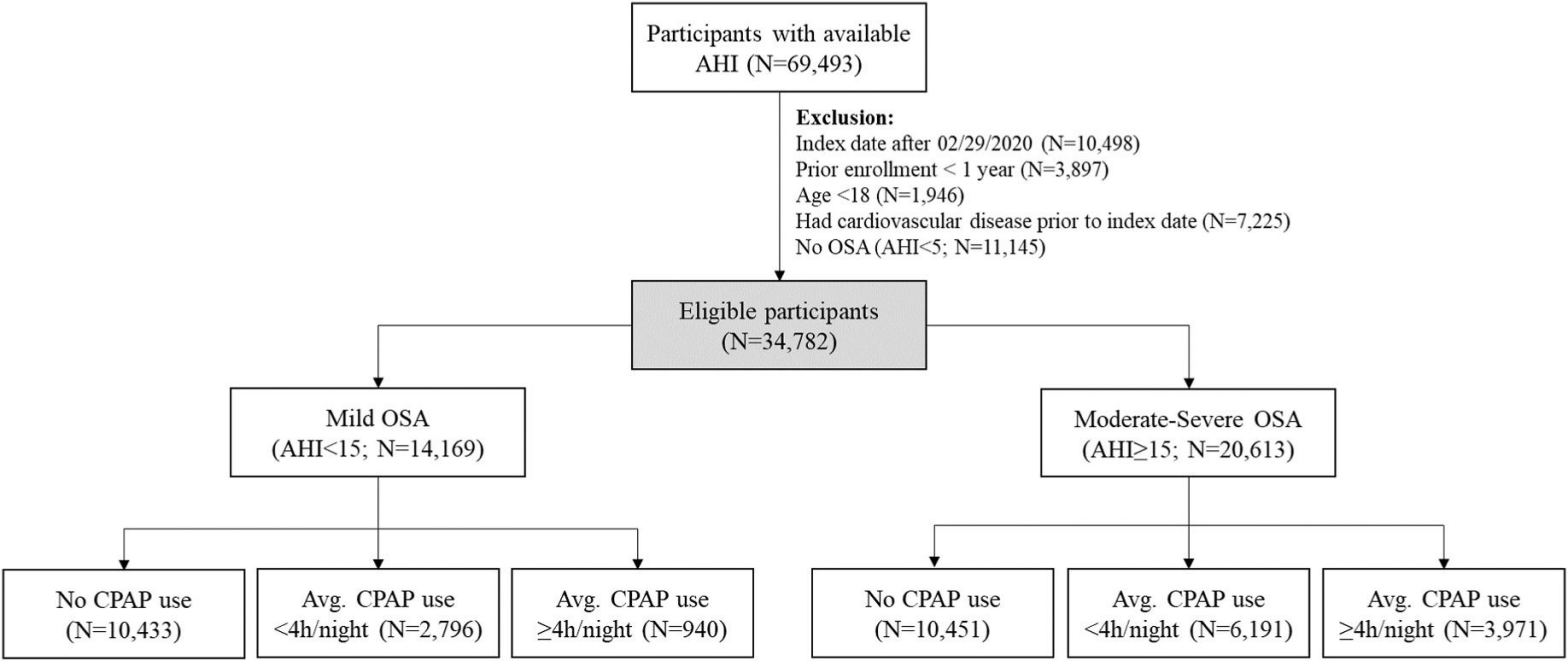
Study flowchart indicating inclusion and exclusion criteria, participant eligibility and groups according to OSA severity and CPAP utilisation. AHI: apnea-hypopnoea index; OSA: obstructive sleep apnea; CPAP: continuous positive airway pressure. Average use corresponds to CPAP utilisation over the exposure period.

Fig. 1 shows the study flowchart summarising eligibility and exclusion criteria, and participant counts in each group. A total of 34,782 adults with a diagnosis of OSA (AHI≥5) were included. Baseline characteristics, follow-up time, and average CPAP use for each exposure group (*No CPAP*, *CPAP* <*4 h/night* and *CPAP* ≥*4 h/night*) are described in Table 1 (in mild OSA) and Table 2 (in moderate-severe OSA).

### 3.1. Clinical and demographic characteristics

Among both mild and moderate-severe OSA participants, those using CPAP ≥4 h/night were older compared to the those not using CPAP or using CPAP <4 h/night, at baseline. They also had higher AHI and were more likely to be men, White, live in geographic areas with >75 % of the population with at least high-school degree, and use lipid lowering medications (Table 1; Table 2). Moderate-severe OSA participants using CPAP ≥4 h/night were more likely to use antihypertensives, less likely to be current smokers and had lower total cholesterol (Table 2). No differences in proportion of the categories of the CCI and CPAP use group were observed (Table 1; Table 2). The mean (± standard deviation) CPAP use of mild OSA participants using CPAP <4 h/night was 1.2 ± 1.2 h and for those using CPAP ≥4 h/night it was 5.7 ± 1.1 h. The mean (± standard deviation) CPAP use of moderate-severe OSA participants using CPAP <4 h/night was 1.5 ± 1.2 h and for those using CPAP ≥4 h/night it was 5.9 ± 1.2 h. The mean follow-up time for those that did not initiate CPAP was 253.6 days, those with CPAP use <4h was 304.0 days, and for those with CPAP use≥4h was 314.5 days. Baseline influenza vaccination rates and appointment no-show rates were similar across CPAP groups in both the mild and moderate-severe OSA cohorts; thus, these metrics did not indicate evidence of healthy-adherer bias.

### 3.2. MACE incidence rates and survival curves among CPAP exposure groups

Unadjusted MACE incidence rates (per 1000 person-years) among CPAP exposure groups are shown in Table 3. In participants with mild OSA, we did not find significant differences in incidence rates among those that did not use CPAP (3.85; 95%CI = 2.76–5.22) and those that used CPAP <4 h/night (3.33; 95%CI = 1.66–5.96) or ≥4 h/night (4.46; 95%CI = 1.45–10.40). However, MACE incidence rates in moderate-severe OSA participants using CPAP ≥4 h/night (3.61; 95%CI = 2.10–5.78) were significantly lower than those that did not use CPAP (7.51; 95%CI = 5.90–9.41). No differences in event rates between participants using CPAP <4 h/night (4.23; 95%CI = 2.85–6.04) and CPAP ≥4 h/night were observed among participants with moderate-severe OSA.

**Fig. 2.**
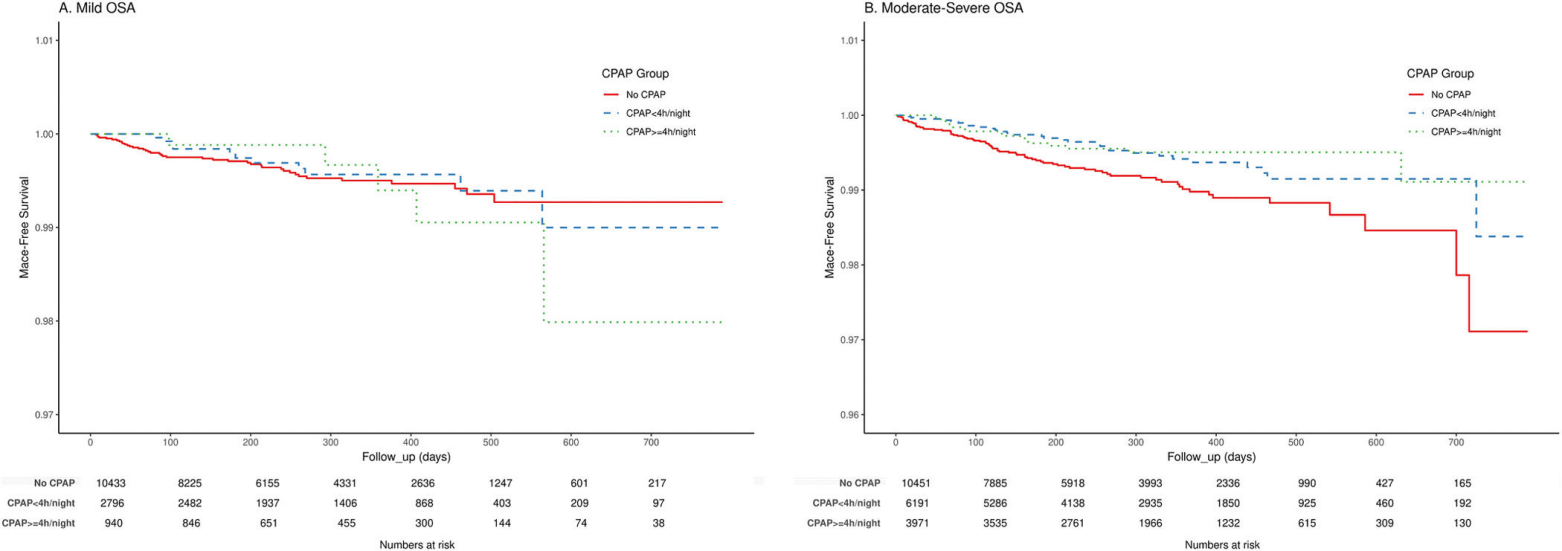
Kaplan-Meier survival analyses describing the association between CPAP use (*no CPAP*, *CPAP* <*4 h/night* and *CPAP* ≥*4 h/night*) and MACE incidence among patients with mild (**A**) and moderate-severe OSA (**B**).

Results of Kaplan-Meier analyses describing the probability of being free of MACE for no CPAP, CPAP <4 h/night and CPAP ≥4 h/night over time are shown in Fig. 2. In unadjusted analyses among participants with mild OSA, we found no evidence of association between CPAP exposure groups and probabilities of being free of MACE (log-rank test p = 0.855; Fig. 2A). Among those with moderate-severe OSA, however, we found a significant association between lower CPAP utilisation and MACE incidence (log-rank test p = 0.0028; Fig. 2B). Moderate-severe OSA participants not on CPAP were the most likely to experience MACE during follow-up.

### 3.3. Moderate-severe OSA participants using CPAP have lower MACE risk compared to those untreated

To determine the strength of the association between CPAP use and MACE incidence among participants with mild or moderate-severe OSA, we used Cox proportional hazards regression analyses adjusted for relevant demographics, lifestyle and cardiovascular risk factors (Table 3). Among participants with mild OSA, those who did not use CPAP had similar MACE incidence as those who used it (CPAP <4 h/night, HR [95 % CI] =0.90 [0.44–1.81], p =0.760) or CPAP ≥4 h/night, HR [95 % CI] 1.15 [0.44–2.98], p = 0.776). However, among participants with moderate-severe OSA, those who used CPAP had significantly lower MACE incidence than those who did not (CPAP <4 h/night: HR [95 % CI] = 0.60 [0.38–0.95]; p = 0.029 and CPAP≥4 h/night: HR [95 % CI] = 0.46 [0.26–0.81]; p = 0.008). Results of inverse probability of treatment weighted Cox proportional hazards models assessing the effect of PAP use on MACE revealed similar results. Among mild OSA, no significant differences in MACE incidence were observed across CPAP exposure groups (CPAP <4 h/night: HR [95 % CI] = 0.77 [0.35–1.69], p = 0.516; CPAP ≥4 h/night: HR [95 % CI] 1.03 [0.34–2.85], p = 0.952). In contrast, moderate-severe OSA participants that used CPAP had significantly lower MACE incidence than those who did not (CPAP <4 h/night: HR [95 % CI] = 0.53 [0.34–0.82]; p = 0.004 and CPAP≥4 h/night: HR [95 % CI] = 0.46 [0.27–0.77]; p = 0.004). These results indicate that, compared to moderate-severe OSA participants not using CPAP, those using CPAP were roughly twice as likely to not experience MACE during follow-up.

To assess whether there was a linear relationship between increased hours of CPAP use and lower MACE incidence risk, we evaluated a Cox proportion hazards model including continuous CPAP use (in hours) as the independent variable. Increased countinuous CPAP use was associated with lower MACE incidence risk in moderate-severe OSA (HR [95 % CI] = 0.90 [0.82–0.98]; p = 0.021), but not on those with mild OSA (HR [95 % CI] = 1.04 [0.91–1.20]; p = 0.540). There was no evidence of significant quadratic effects of hours of CPAP use in both mild and moderate-severe OSA participants.

## 4. Discussion

This study evaluated the association between CPAP therapy and MACE in a longitudinal clinical cohort of adults with OSA at KPSC, a large healthcare system in the United States. We leveraged a cutting-edge CPAP telemonitoring integration framework to allow accurate linkage between CPAP use, relevant diagnostic sleep study information, and MACE outcomes in ‘real-world’ data from KPSC. Thus, the study is representative of patients seeking OSA care from a specialised sleep clinic. We demonstrated that among participants with moderate-severe OSA, those not using CPAP are two times more likely than those using CPAP to experience a MACE event during follow-up, independent of cardiovascular risk factors. Our findings suggest that CPAP therapy might have a positive impact on cardiovascular risk prevention among clinical patients at elevated risk, e.g., those with moderate-severe OSA.

Several pathophysiological mechanisms might explain the relationship between OSA and cardiovascular diseases. Sympathetic activation, endothelial dysfunction, oxidative stress, systemic inflammation, cardiac remodeling due to hypoxemia, intrathoracic pressure swings and blood pressure changes [32] occur as a result of OSA. We have recently demonstrated a dose-response association between OSA severity and less variation in heart rate variability during wakefulness [33], which has also been reported as an independent risk factor for MI [34–36] and cardiovascular mortality [36]. These overlapping mechanisms indicate that therapies targeting OSA, such as CPAP, are likely to be beneficial for cardiovascular health and preventing cardiovascular outcomes.

Meta-analytical evidence from RCTs established that CPAP therapy reduces systolic and diastolic blood pressure [12], especially in patients with resistant hypertension [13]. CPAP therapy also offers a favorable effect on insulin resistance [37] and prevents metabolic disturbances in patients with insulin resistance [38]. Furthermore, CPAP therapy has positive effects on long-term survival in patients with ischemic stroke and moderate-severe OSA [39]. A prospective cohort study found that CPAP was associated with a lower rate of fatal and non-fatal CV events over a 10-year follow-up, compared to untreated severe OSA [17]. Furthermore, an observational study using the French National Health Insurance Reimbusrement System Database (N = 176,014) suggests that in adults, CPAP termination (e.g., cessation of CPAP reimbursement claims) was associated with increased all-cause mortality and incident heart failure [40]. These results also corroborate our own recent findings in Medicare beneficiaries in the Central U.S. (N = 888,835), which reported lower all-cause mortality and MACE risk among those with evidence of CPAP initiation or greater CPAP utilisation in the first year, using claims data [41]. A recent meta-analysis of both randomised and non-ramdomised studies which included both studies also confirmed these results, although mostly driven by the non-randomised studies [42].

RCTs assessing the effect of CPAP on fatal and non-fatal cardiovascular outcomes [21–23] found that CPAP does not prevent them. Critical assessment of existing RCTs and the role of alternative study designs to address some of the most relevant limitations related to generalisability of these trials have been published elsewhere [25], and extensively discussed [43,44]. Notably, patient populations enrolled in RCTs are not representative of patients observed in sleep clinics seeking care for their OSA [26]. Enrolled participants had established cardiovascular disease and, therefore, studies would be applicable as secondary prevention, which does not always reflect the primary population of OSA patients observed in the sleep clinic. Furthermore, excessive daytime sleepiness was an exclusion criteria used in recents trials, primarily due to the ethical concerns of randomising participants that are excessively sleepy to control arms [25]. OSA patients that are excessively sleep represent roughly 40 % of all moderate-severe OSA patients in a clinical cohort from international sleep centers [45], and are also at the greatest cardiovascular risk [46–49]. Finally, CPAP adherence observed in RCTs was generally low when compared to clinical populations [50]. High-lighting the impact of low adherence on study conclusions, secondary analysis restricted to patients with increased adherence showed CPAP benefits that are consistent with what was observed by cohort studies [21,22], yet were underpowered to detect effects as significant [25]. Given these critical assessments, it may be argued that recent RCTs have answered the more specific question of whether suboptimal CPAP therapy in less symptomatic adults prevents secondary cardiovascular outcomes, rather than the overarching question of whether treatment with CPAP has cardiovascular benefits [25].

Although observational, our current study is representative of a real-world clinical cohort of adults seeking care for OSA and, therefore, more amenable to identify cardiovascular benefits of CPAP therapy consistent with prior epidemiological evidence in similar samples. Moreover, we explored a robust framework based on inverse probability of treatment weights [51] derived from propensity score models estimating a participants propensity to use CPAP. We also included participants without evidence of major cardiovascular outcomes in the year prior to OSA diagnosis, which constituted the majority of our initial cohort (89.6 %; Fig. 1) prior to applying any inclusion/exclusion criteria. Furthermore, although sleepiness was not directly assessed by our study (a noted limitation), our clinical sample is expected to include adults with OSA with all degrees of sleepiness based on what is routinely seen in sleep clinics [45,52]. Recent observations that individuals with moderate-severe OSA and excessive sleepiness are at particularly increased risk for cardiovascular-related events [46,49] suggest that the benefits of CPAP observed in our clinical sample would be even stronger if restricted to excessively sleepy participants. These associations seem to be significant when addressing outcomes defined as combined fatal or non-fatal cardiovascular events, while when looking specifically at fatal cardiovascular events, excessively sleepiness may play a more limited role [46,53]. This is an important future consideration when evaluating the benefits of CPAP therapy in at-risk patients with OSA.

Studies have also reported that certain characteristics of sleep quality and quantity are associated with cardiovascular complications (for review, see Ref. [54]). Short sleep duration has been suggested to be a causal risk factor for MI [55]. Moreover, shorter sleep duration [56], longer sleep onset latency [57], and more frequent nighttime awakening [57] have all been associated with increased odds of atrial fibrillation, when adjusted for sleep-disordered breathing. Furthermore, insomnia diagnosis was a predictor of a subsequent diagnosis of atrial fibrillation [57]. Comorbid insomnia with OSA (e.g., COMISA) is highly prevalent (30–50 %) [58], and patients with the comorbid syndrome are less likely to accept CPAP therapy, resulting in a lower highly CPAP use [59]. Assessment of clinical diagnosis of insomnia utilising EHR-based cohorts is challenging and might require the application of natural language processing methods to derive reliable diagnostic information. Data abstraction in sleep medicine is currently suboptimal for EHR-based cohorts [60], and specific definitions based on diagnostics tests, as performed in the current study, are preferred. Deep phenotypying of sleep disorders using EHR data, by integrating different sources of patient-generated and diagnostic data, should be the focus of future investigatons.

### 4.1. Strengths and limitations

The current study has several strengths, including being among the largest studies linking daily CPAP telemonitoring data with cardiovascular outcomes of public health importance. We include a wide range of covariates in the analysis, particularly a well curated set of factors that capture healthy user bias, allowing us to provide more accurate estimates of treatment effects. We also apply robust causal inference methods, confirming associations observed in covariate-adjusted models. Results within the population of this study are more likely to generalise to adults with OSA typically seen in sleep clinics, particularly when contrasted with recent RCTs. The integrated KPSC healthcare system is closed with minimal distinction between primary and secondary care. In addition, out-of-network claims for KPSC members are captured in the system. This contributes to minimising the likelihood that outcomes would be recorded in out-of-network health systems and not captured by our study. We were careful to not allow gaps >90 days in insurance coverage, which would minimise loss of follow-up or missing outcome data even further.

Our study also has limitations. First, we were not able to determine OSA symptom presentation due to the lack of readily available participant-reported symptom data. Moderate-severe OSA patients that are excessively sleepy are at the highest risk for incident cardiovascular events [46,49], although not cardiovascular mortality [46,53]. While speculative, it is likely this subgroup composed a significant proportion of our cohort, and may be driving the observed associations with increased cardiovascular risk among the untreated. Second, our study relied on EHR-based definition of outcomes, as such, ascertainment was performed via computable phenotyping. However, these outcomes were validated within KPSC and used in prior investigations [61–67], which supports their internal validity. Third, we used a conventional, yet admittedly arbitrary definition of CPAP use to determine adherence (≥4 h/night). We acknowledge that this definition is not well rationalised, and stems from current Centers for Medicare and Medicaid Services criteria to support insurance coverage of CPAP therapy [68]. While this definition facilitates comparisons to other existing studies, future investigations should evaluate whether there are more optimal definitions of adherence with respect to cardiovascular risk. To mitigate this limitation, we presented analyses on the association of CPAP use as a continuous variable, which supported the results on the categorical definition used in our primary analysis. Fourth, our study assumed that participants without evidence of CPAP use were not using CPAP therapy and therefore representative of an untreated population. However, it is feasible that a small proportion may have been using CPAP but had missing adherence data in our system, or that they could have been using other OSA therapies (i.e., oral devices or hypoglossal nerve stimulation). Importantly, the presence of participants that are treated for OSA within the no CPAP group will bias results towards the null hypothesis of no CPAP benefit on cardiovascular endpoints. Fifth, while we included a number of relevant covariates in our adjusted analyses, it is possible that additional unmeasured confounding could have influenced our results. However, we were careful to include potential confounders related to healthy adherer and user bias, such as baseline adherence to influenza vaccination and medical appointment no-show rates. Sixth, our study also had a relatively short follow-up time of approximately 9–10 months on average, which resulted in few cardiovascular events in longitudinal analyses and prevented us from looking at different types of events separately. While our large sample size resulted in adequate statistical power to identify the described effects as statistically significant despite the lower number of events, longer follow-up time will provide more accurate effect estimates. Large-scale CPAP telemonitoring is innovative, but only widely available in recent years [27]. Moreover, we made the decision to only include participants with diagnostic sleep study date on or prior to February 29th^,^ 2020, minimising the potential effects of changes in healthcare operations and variables under study due to the Coronavirus Disease 2019 pandemic. The inclusion time-frame also proceeded an unforseen major recall of Philips CPAP devices in 2021, affecting thousands of KPSC members, which would have dramatically affected our exposure definitions by decreasing the average CPAP utilisation for those using such devices.

## 5. Conclusion

Our analysis in a large observational clinical cohort of adults from a sleep clinic found that among moderate-severe OSA participants, those not using CPAP were two times more likely than those using CPAP to experience a MACE during follow-up. These results suggest that treatment of moderate-severe OSA with CPAP may have a positive impact on cardiovascular disease prevention, and also have implications for future studies of the long-term benefits of therapy.

## Supplementary Material

Multimedia component 2

Multimedia component 1

Multimedia component 3

## Figures and Tables

**Table 1:** Clinical and demographic characteristics of sample of patients with mild OSA according to CPAP use.

Variable	Category	Mild OSA (N = 14,169)			
	
		No CPAP (N = 10,433)	CPAP <4 h/night (N = 2796)	CPAP ≥4 h/night (N = 940)	p

**Age**, years		51.3 ± 14.0	50.6 ± 13.5	54.1 ± 13.3	<0.001^a,b,c^

**Sex**	Men	5345 (51.2 %)	1501 (53.7 %)	555 (59 %)	<0.001^a,b,c^
	Women	5088 (48.8 %)	1295 (46.3 %)	385 (41 %)	
					
**Race/Ethnicity**	Hispanic	3799 (36.4 %)	1007 (36 %)	262 (27.9 %)	<0.001 ^b,c^
	Non-Hispanic Asian or Pacific Islander	1085 (10.4 %)	275 (9.8 %)	68 (7.2 %)	
	Non-Hispanic Black	923 (8.8 %)	292 (10.4 %)	51 (5.4 %)	
	Non-Hispanic Other/Unknown	428 (4.1 %)	107 (3.8 %)	45 (4.8 %)	
	Non-Hispanic White	4198 (40.2 %)	1115 (39.9 %)	514 (54.7 %)	
					
**Marital status**	Not living with partner	4727 (45.3 %)	1232 (44.1 %)	359 (38.2 %)	<0.001 ^b,c^
	Living with partner	5706 (54.7 %)	1564 (55.9 %)	581 (61.8 %)	
					
**Educational level**, geocoded % of high school degree and above	0–50 %	247 (2.4 %)	57 (2.0 %)	12 (1.4 %)	0.011 ^b,c^
	51–75 %	2176 (20.9 %)	595 (21.3 %)	164 (17.5 %)	
	76–100 %	7990 (76.7 %)	2139 (76.6 %)	763 (81.3 %)	
					
**Median household income**, geocoded		$80.3k [59.3k-105.6k]	$78.1k [57.1k-100.7k]	$81.7k [61.4k-104.1k]	<0.001^a,c^

**BMI**, kg/m^2^		32.8 ± 7.3	33.0 ± 7.3	32.9 ± 6.9	0.291

**AHI**, events/h		9.2 ± 2.9	9.7 ± 2.9	10.1 ± 2.9	<0.001^a,b,c^

**Charlson comorbidity index**	0	6225 (59.7 %)	1652 (59.1 %)	537 (57.1 %)	0.415

	1–2	3364 (32.2 %)	931 (33.3 %)	328 (34.9 %)	
			
	>2	844 (8.1 %)	213 (7.6 %)	75 (8 %)	
					
**Antihypertensives use**	Yes	3989 (38.2 %)	1098 (39.3 %)	403 (42.9 %)	0.016 ^b^

**Systolic blood pressure**, mmHg		125.6 ± 12.7	125.6 ± 12.8	126.2 ± 12.2	0.388

**Lipid-lowering medications use**	Yes	2964 (28.4 %)	818 (29.3 %)	357 (38 %)	<0.001 ^b,c^

**Total cholesterol**, mg/dL		185.3 ± 40.3	183.6 ± 41.1	181.3 ± 39.5	0.001^a,b^

**HDL-cholesterol**, mg/dL		49.6 ± 13.2	49.0 ± 12.8	49.1 ± 12.9	0.037^a^

**Smoking status**	Never	7219 (72.9 %)	1875 (70.7 %)	625 (70.7 %)	0.061
	Former	2188 (21.4 %)	622 (23.5 %)	215 (24.3 %)	
	Current	565 (5.4 %)	154 (5.8 %)	44 (5.0 %)	
					
**Physical activity**, h/week		1.0 [0.0–3.0]	1.0 [0.0–2.7]	1.0 [0.0–3.0]	0.620

**Baseline Influenza vaccination rate**	Yes	8100 (77.6 %)	2189 (78.3 %)	759 (80.7 %)	0.080

**Baseline Appointment No-show rate**	0 %	9439 (90.5 %)	2482 (88.8 %)	863 (91.8 %)	0.004^a,c^

	0–10 %	520 (5.0 %)	183 (6.5 %)	36 (3.8 %)	
			
	≥10 %	474 (4.5 %)	131 (4.7 %)	41 (4.4 %)	
					
**Follow-up time**, days		271.0 ± 184.97	313.5 ± 178.01	316.7 ± 180.88	<0.001^a,b^

**Average CPAP use**, h/night		0.0 ± 0.0	1.2 ± 1.2	5.7 ± 1.1	<0.001^a,b,c^

Average CPAP use was defined based on exposure period (index date to censoring). Categorical variables as represented as N (%). Median household income and physical activity are represented as median [interquartile range]; all other continuous variables are represented as mean ± standard deviation. Comparisions among groups were performed using Kruskal-Wallis or chi-squared tests. Significant (p < 0.05) pairwise comparisons indicated as:

a No CPAP vs. CPAP <4 h/night;

b No CPAP vs. CPAP ≥4 h/night;

c CPAP <4 h/night vs. CPAP ≥4 h/night. Abbreviations: AHI: apnea-hypopnoea index; BMI: body mass index; CPAP: continuous positive airway pressure; HDL: high-density lipoprotein.

**Table 2:** Clinical and demographic characteristics of sample of patients with moderate-severe OSA according to CPAP use.

Variable	Category	Moderate-severe OSA (N = 20,613)			
	
		No CPAP (N = 10,451)	CPAP <4 h/night (N = 6191)	CPAP ≥4 h/night (N = 3961)	p

**Age**, years		52.6 ± 13.5	51.0 ± 13.2	53.5 ± 13.0	<0.001^a,b,c^

**Sex**	Men	6925 (66.3 %)	4063 (65.6 %)	2760 (69.5 %)	<0.001^a,b,c^
	Women	3526 (33.7 %)	2128 (34.4 %)	1211 (30.5 %)	
					
**Race/Ethnicity**	Hispanic	4057 (38.8 %)	2463 (39.8 %)	1246 (31.4 %)	<0.001^a,b,c^
	Non-Hispanic Asian or Pacific Islander	1236 (11.8 %)	710 (11.5 %)	394 (9.9 %)	
	Non-Hispanic Black	838 (8 %)	690 (11.1 %)	270 (6.8 %)	
	Non-Hispanic Other/Unknown	449 (4.3 %)	262 (4.2 %)	170 (4.3 %)	
	Non-Hispanic White	3871 (37 %)	2066 (33.4 %)	1891 (47.6 %)	
					
**Marital status**	Not living with partner	4724 (45.2 %)	2729 (44.1 %)	1625 (40.9 %)	<0.001 ^b,c^

	Living with partner	5727 (54.8 %)	3462 (55.9 %)	2346 (59.1 %)	
					
**Educational level**, geocoded % of high school degree and above	0–50 %	287 (2.8 %)	171 (2.8 %)	78 (2.0 %)	<0.001 ^b,c^
	51–75 %	2415 (23.1 %)	1526 (24.7 %)	795 (20.0 %)	
	76–100 %	7733 (74.1 %)	4488 (72.6 %)	3095 (78.0 %)	
					
**Median household income**, geocoded		$78.0k [57.9k-101.8k]	$75.3k [55.6k-98.8k]	$78.8k [60.3k-102.5k]	<0.001^a,b,c^

**BMI**, kg/m^2^		35.4 ± 8.0	36.2 ± 8.2	36.3 ± 7.9	<0.001^a,b^

**AHI**, events/h		36.9 ± 22.2	39.9 ± 22.6	46.0 ± 24.3	<0.001^a,b,c^

**Charlson comorbidity index**	0	5835 (55.8 %)	3426 (55.3 %)	2226 (56.1 %)	0.432

	1–2	3652 (34.9 %)	2205 (35.6 %)	1416 (35.7 %)	
			
	>2	964 (9.2 %)	560 (9 %)	329 (8.3 %)	
					
**Antihypertensives use**	Yes	4945 (47.3 %)	2942 (47.5 %)	2065 (52 %)	<0.001 ^b,c^

**Systolic blood pressure**, mmHg		128.3 ± 12.7	128.5 ± 12.3	129.1 ± 12.6	0.004 ^b,c^

**Lipid-lowering medications use**	Yes	3640 (34.8 %)	2093 (33.8 %)	1549 (39 %)	<0.001 ^b,c^

**Total cholesterol**, mg/dL		183.7 ± 42.2	183.5 ± 40.7	179.7 ± 41.1	<0.001 ^b,c^

**HDL-cholesterol**, mg/dL		46.4 ± 12.1	46.0 ± 11.8	45.8 ± 11.8	0.001 ^b^

**Smoking status**	Never	6838 (69.7 %)	3948 (67.9 %)	2575 (68.9 %)	0.001 ^b,c^
	Former	2258 (23.0 %)	1400 (24.1 %)	934 (25.0 %)	
	Current	714 (7.3 %)	464 (8.0 %)	228 (6.1 %)	
					
**Physical activity**, h/week		0.5 [0.0–2.5]	0.3 [0.0–2.3]	0.5 [0.0–2.5]	0.081

**Baseline Influenza vaccination rate**	Yes	7993 (76.5 %)	4634 (74.9 %)	3089 (77.8 %)	0.002^a,c^

**Baseline Appointment No-show rate**	0 %	9392 (89.9 %)	5560 (89.8 %)	3667 (92.3 %)	<0.001 ^b,c^

	0–10 %	514 (4.9 %)	300 (4.8 %)	140 (3.5 %)	
			
	≥10 %	545 (5.2 %)	331 (5.3 %)	164 (4.1 %)	
					
**Follow-up time**, days		253.6 (179.68)	304.0 (183.50)	314.5 (178.82)	<0.001^a,b,c^

**Average CPAP use**, h/night		0.0 ± 0.0	1.5 ± 1.2	5.9 ± 1.2	<0.001^a,b,c^

Average CPAP use was defined based on exposure period (index date to censoring). Categorical variables as represented as N (%). Median household income and physical activity are represented as median [interquartile range]; all other continuous variables are represented as mean ± standard deviation. Comparisions among groups were performed using Kruskal-Wallis or chi-squared tests. Significant (p < 0.05) pairwise comparisons indicated as:

a No CPAP vs. CPAP <4 h/night;

b No CPAP vs. CPAP ≥4 h/night;

c CPAP <4 h/night vs. CPAP ≥4 h/night. Abbreviations: AHI: apnea-hypopnoea index; BMI: body mass index; CPAP: continuous positive airway pressure; HDL: high-density lipoprotein.

**Table 3:** Results of the Cox proportional hazards model assessing the association between OSA severity combined with different levels of CPAP use and MACE incidence, compared to individuals of the same OSA severity but not using CPAP.

Exposure groups		Events	Person-years	MACE incidence rate (95 % CI)	Hazard Ratio (95 % CI)			
OSA severity	CPAP use				Adjusted model	p	IPTW model	p

Mild OSA	No CPAP	41	10,661	3.85 (2.76, 5.22)	1.00 (ref)	–	1.00 (ref)	–
	CPAP <4 h/night	11	3305	3.33 (1.66, 5.96)	0.90 (0.44–1.81)	0.760	0.77 (0.35–1.69)	0.516
	CPAP ≥4 h/night	5	1122	4.46 (1.45, 10.40)	1.15 (0.44–2.98)	0.776	1.03 (0.37–2.85)	0.952
								
Moderate-severe OSA	No CPAP	75	9992	7.51 (5.90, 9.41)	1.00 (ref)	–	1.00 (ref)	–
	CPAP <4 h/night	30	7095	4.23 (2.85, 6.04)	**0.60 (0.38–0.95)**	**0.029**	**0.53 (0.35–0.82)**	**0.004**
	CPAP ≥4 h/night	17	4709	3.61 (2.10, 5.78)	**0.46 (0.26–0.81)**	**0.008**	**0.46 (0.28–0.77)**	**0.004**

Event rates are presented per 1000 person-years. Hazard ratios are adjusted for age, sex, race/ethnicity, educational level, household income, BMI, CCI, use of antihypertensives, systolic blood pressure, use of lipid-lowering medications, total cholesterol HDL-cholesterol, smoking status and physical activity. IPTW models further included weights calculated from propensity scores derived from logistic regression of covariates of CPAP use.

OSA severity exposure groups were defined as mild OSA: AHI between 5 and 15 events/h; moderate-severe OSA: AHI≥15 events/h.

Abbreviations: CI: confidence interval; CPAP: continuous positive airway pressure; IPTW: inverse probability of treatment weights; MACE: major adverse cardiovascular events; OSA: obstructive sleep apnea.

## Data Availability

The data underlying this article cannot be shared publicly to protect the privacy of individuals that participated in the study, as stipulated by KPSC.

## Appendix A. Supplementary data

Supplementary data to this article can be found online at https://doi.org/10.1016/j.sleep.2025.108732.
