# Supplementary material for: Positive airway pressure therapy and cardiovascular events in obstructive sleep apnoea: an observational clinical cohort study
Source: Sleep Med. Author manuscript; Available in PMC 2026 May 26. (PMC13203992; doi:10.1016/j.sleep.2025.108732)
Supplement: Multimedia component 1 [file NIHMS2166968-supplement-Multimedia_component_1.docx]

**Title:** Positive Airway Pressure Therapy and Cardiovascular Events in Obstructive Sleep Apnoea: an Observational Clinical Cohort Study

Diego R. Mazzotti, Ph.D, Aiyu Chen, M.P.H., Jaejin An, BPharm, Ph.D., Joanie Chung, M.P.H., Jessica Arguelles, B.Sc., Brendan T. Keenan, M.S., Greg Maislin, M.S., Bruno Saconi, Ph.D., R.N., Alexa J.Watach, Ph.D., R.N., Henry Glick, Ph.D., Samuel T. Kuna, M.D., Allan I. Pack M.B.Ch.B., Ph.D., Amy M. Sawyer, Ph.D., R.N., Dennis Hwang, M.D., Jiaxiao Shi, Ph.D.

**Supplemental Material**

**Supplemental methods**

*Study participants*

This is an observational retrospective analysis of EHR and administrative data from Kaiser Permanente Southern California (KPSC), a large integrated healthcare system in the U.S. serving over 4.6 million members. KPSC provides services to its members through 15 hospitals, more than 200 outpatient facilities and a centralised laboratory, including 10 sleep disorders clinics. Historical data indicate a large member retention rate at KPSC (e.g., 87% for year 2021).

Adults (age ≥18 years) referred to one of the Sleep Disorders Clinics at KPSC between January 2018 and February 2020 and with a diagnosis of OSA based on the apnoea-hypopnea index (AHI)≥5 events/hours from a clinical diagnostic sleep study (polysomnography or home sleep apnoea test) were included in the study. The index date was defined as the date of the diagnostic sleep study. To be eligible, participants must have had at least one year of insurance coverage at KPSC prior to sleep study date (index date) and not have had a cardiovascular event (see outcomes definition below) in the year prior to the index date. Participants with gaps greater than 90 days in insurance coverage during their period of exposure (time between index date and censoring) were censored at the first enrollment stop date. The identified participants were followed until the outcome of interest, disenrollment, death, or study end date (August 2020), whichever occurred first. This study was reviewed by the KPSC Institutional Review Board and determined to be minimal risk; thus informed consent requirement was waived.

*OSA diagnosis and severity*

Polysomnography (Sandman, Natus, Inc.) or home-based sleep apnoea tests (Nox T3, Nox Medical; WatchPAT 200/300, Itamar Medical , LTD.) were used to diagnose OSA in the clinical settings. AHI was defined as the number of respiratory events (apnoeas ≥10 seconds and hypopneas ≥10 seconds associated with ≥4% oxygen desaturation) divided by the total sleep time (polysomnography) or total recording time (home sleep studies) in hours, according to criteria established by the American Academy of Sleep Medicine Manual for the Scoring of Sleep and Associated Events ^1^. OSA was defined when the AHI was ≥5 events/hour. OSA severity categories were determined based on the AHI as mild OSA (AHI≥5 and <15 events/hour) and moderate-severe OSA (AHI≥15 events/hour).

*CPAP telemonitoring and exposure definitions*

CPAP utilisation data were obtained via telemonitoring with cloud-based services provided by major CPAP vendors (i.e., Resmed Airview and Philips EncoreAnywhere). We utilised an established CPAP telemonitoring data integration framework that consists of application programming interface connections with vendors and data exchange via Health Level 7^®^ interfaces, provided through the Care Management module of the Somnoware system (Somnoware Healthcare Systems, Inc, California, U.S.). Daily CPAP utilisation data (i.e., hours of use/night) were averaged across the exposure period (from index date through censoring) to determine three exposure groups: no CPAP (average utilisation of zero or not available), CPAP use <4 hours/night, and CPAP use ≥4 hours/night.

*Cardiovascular outcomes*

Our primary major adverse cardiovascular events (MACE) endpoint was a composite of the first occurrence of myocardial infarction (MI), stroke, unstable angina, heart failure or cardiovascular death. We used previously validated EHR algorithms (computable phenotypes) to identify these events. MI was identified by principal hospital discharge diagnoses with International Classification of Diseases (ICD)-9 codes 410.x0, 410.x1 or ICD-10 codes I21.x ^2^. Stroke was identified by principal and secondary hospital discharge diagnoses with ICD-9 codes 430, 431, 432.x, 433.x1, 434.x1, 436.xx, or ICD-10 I60.9, I61.x, I62.x, I63.x, I67.89 ^3,4^. Unstable angina was identified by principal ICD-9 codes 411.1 or ICD-10 codes I20, I24.0, I24.1, I24.8, I24.9, I25.110, I25.7x0-I25.7x0, or principal ICD-9 codes of 414.xx or ICD-10 codes I25.111, I25.118, I25.119, I25.7x1, I25.7x8, I25.7x9, I25.3, I25.41, I25.41, I25.82, I25.84 and having 411.x as a secondary code ^5^; heart failure was identified as either having a hospitalisation with a principal discharge diagnosis of heart failure and/or having ≥3 ambulatory visits coded for heart failure with at least one visit being with a cardiologist (ICD-9 codes 402.x, 404.x, 428, 428.x, 398.91; ICD 10 codes: I09.81, I11.0, I13.0, I13.2, I50.x, I97.x) ^6-8^. Finally, cardiovascular death was defined as death from coronary heart disease, identified using ICD-10 codes (I20-I25) from hospital records, and death files from state and federal sources ^9^.

*Covariates*

Relevant demographic and clinical characteristics were obtained from the KPSC EHR at baseline (at the time of OSA diagnosis) and used as covariates in the study. These included: age, sex, race/ethnicity (Hispanic, Non-Hispanic Asian/Pacific Islander, Non-Hispanic Black, Non-Hispanic Others/Unknown, and Non-Hispanic White), marital status (living with partner or not), educational level based on geocoded proportion of population with high school degree or above (0-50%, 51-75% and 75-100%), geocoded median household income (continuous, presented as thousands of U.S. dollars), body mass index (BMI, in kg/m^2^), Charlson Comorbidity Index (CCI; stratified as 0, 1-2 or >2 ^10,11^), prescriptions or dispensing of anti-hypertensives (e.g., beta-blockers, calcium-channel blockers, diuretics; see **Supplemental Table 1** for list of specific medications), systolic blood pressure (measured closest to index date), prescriptions or dispensing of lipid-lowering medications (e.g., statins; see **Supplemental Table 1** for list of specific medications), total cholesterol (mg/dL), high-density lipoprotein (HDL) cholesterol (mg/dL), smoking status (never, former and current), and physical activity status based on exercise vital sign, a validated index collected during each encounter designed to identify patients not meeting physical activity recommendations ^12^. In addition, to minimise the possibility of healthy user and healthy adherer bias on the effect of CPAP therapy on MACE risk ^13^, we included a variable representing whether participants had their flu shot in the year prior to the index date and a variables indicating whether participants cancelled medical appointments (e.g., ‘no-show’ rate) categorised as 0%, <10% and ≥10% no-show rate.

*Statistical analyses*

Baseline demographic and clinical characteristics were described among exposure groups using counts and percentages for categorical data and mean and standard deviation (SD) or median and interquartile range for continuous data. Univariate associations between demographic and clinical variables with OSA diagnosis, severity and CPAP exposure groups were performed using chi-squared tests or Fisher’s exact tests (for categorical data), or Kruskal-Wallis’ test (for continuous data). We assessed the association between CPAP utilisation (*no CPAP, CPAP <4 hours/night* and *CPAP ≥4 hours/night*) and incidence of MACE, separately among those with mild OSA (AHI≥5 and <15) and moderate-severe OSA (AHI≥15). Kaplan-Meier survival analysis and the log-rank test were used to compare survival curves among different CPAP utilisation groups. Covariate-adjusted Cox proportional hazards models were used to evaluate associations between CPAP use and MACE, using the *no CPAP* group as the reference category, with complementary analyses using average daily hours of CPAP use with and without quadradic terms (e.g., *CPAP hours* * *CPAP hours*) as the exposure. Further analyses to explore causal inference of the effect of CPAP use on MACE using inverse probability of treatment weighting were also performed. Propensity score (PS) on the likelihood of any CPAP use (<4h or ≥4h/night) were estimated using logistic regression with study covariates for mild and moderate-severe OSA separately. Standardised mean differences were used to check the balance of each characteristics between the two groups, and differences <0.1 were considered as good balance. Inverse probability of treatment weights (1 for the CPAP users and PS/(1-PS) for the non-users) were used together with the CPAP exposure groups in weighted Cox proportional hazards regression models. In mild OSA, the propensity score model using main effects only was chosen as the one minimising standardised mean differences in covariates between exposure groups after weighing, while in moderate-severe OSA, the propensity score model using main effects plus AHI squared was chosen. Results are reported as hazard ratios (HR) and 95% confidence intervals, representing estimates of the average treatment effect on treated. Results with p<0.05 were considered statistically significant. Analyses were done using SAS (v.9.4; SAS Institute).

**Supplemental Tables**

**Supplemental Table 1:** List of medications included in the definition of anti-hypertensives and lipid-lowering medications according to the first 4 digits of Generic Product Identifier (GPI) hierarchical classification system.

|  | | |
| --- | --- | --- |
| **Definition** | **GPI (first 4 digits)** | **Class** |
| Anti-hypertensive | 3310 | Beta-Blockers, nonselective |
|  | 3320 | Beta-Blockers, cardioselective |
|  | 3330 | Alpha-Beta-Blockers |
|  | 3400 | Calcium channel blockers |
|  | 3610 | ACE Inhibitors |
|  | 3615 | ARBs |
|  | 3617 | Direct renin inhibitors |
|  | 3620 | Antiadrenergics |
|  | 3625 | Selective aldosterone receptor antagonists |
|  | 3630 | Agents for pheochromocytoma |
|  | 3640 | Vasodilators |
|  | 3660 | Antihypertensives, miscellaneous |
|  | 3699 | Antihypertensive combinations |
|  | 3720 | Loop diuretics |
|  | 3750 | Potassium-sparing diuretics |
|  | 3760 | Thiazides and thiazidelike diuretics |
|  | 3799 | Diuretic combinations |
|  | 4099 | Cardiovascular agents combinations |
| Lipid-lowering | 3940 | HMG-CoA reductase inhibitors |
|  | 3999 | Antihyperlipidemic combinations |
| Abbreviations: ACE: angiotensin-converting enzyme; ARBs: Angiotensin II Receptor Blockers; HMG-CoA: 3-hydroxy-3-methylglutaryl coenzyme A | | |

**Supplemental Table 2:** Standardised mean differences (SMD) of covariates between continuous positive airway pressure (CPAP) use groups (*no CPAP* vs. *any CPAP*) after inverse probability of treatment weighting. All SMD are below 0.1, suggesting adequate covariate balance after weithing.

| **Covariate** | **SMD** | |
| --- | --- | --- |
|  | **Mild OSA** | **Moderate-severe OSA** |
| Age | -0.058 | -0.041 |
| Sex | 0.04 | 0.041 |
| Race/Ethnicity | 0.057 | 0.077 |
| Marital status | -0.025 | -0.02 |
| Educational level | 0.012 | 0.009 |
| Median household income | -0.003 | 0.001 |
| Body mass index | 0 | -0.008 |
| Apnoea-hypopnea index | -0.009 | 0.004 |
| Charlson comorbidity index | 0.056 | 0.043 |
| Anti-hypertensives | -0.033 | -0.037 |
| Systolic blood pressure | -0.001 | 0 |
| Lipid-lowering medication | -0.03 | -0.033 |
| Total Cholesterol | 0.01 | 0.01 |
| HDL-cholesterol | -0.002 | -0.001 |
| Smoking status | 0.023 | 0.019 |
| Physical activity | 0.008 | 0.007 |
| Flu shots on prior year | -0.047 | -0.046 |
| No-show rate | 0.023 | 0.032 |

**References**

1. Berry RB, Brooks R, Gamaldo C*, et al.* AASM Scoring Manual Updates for 2017 (Version 2.4). *J Clin Sleep Med* 2017;**13**:665-666. doi: 10.5664/jcsm.6576

2. Reynolds K, Go AS, Leong TK*, et al.* Trends in Incidence of Hospitalized Acute Myocardial Infarction in the Cardiovascular Research Network (CVRN). *The American Journal of Medicine* 2017;**130**:317-327. doi: 10.1016/j.amjmed.2016.09.014

3. Singer DE, Chang Y, Borowsky LH*, et al.* A New Risk Scheme to Predict Ischemic Stroke and Other Thromboembolism in Atrial Fibrillation: The ATRIA Study Stroke Risk Score. *Journal of the American Heart Association* 2013;**2**. doi: 10.1161/jaha.113.000250

4. Go AS, Hylek EM, Chang Y*, et al.* Anticoagulation Therapy for Stroke Prevention in Atrial Fibrillation. *Jama* 2003;**290**. doi: 10.1001/jama.290.20.2685

5. Sidney S, Sorel M, Quesenberry CP*, et al.* COPD and Incident Cardiovascular Disease Hospitalizations and Mortality: Kaiser Permanente Medical Care Program. *Chest* 2005;**128**:2068-2075. doi: 10.1378/chest.128.4.2068

6. Go AS, Yang J, Ackerson LM*, et al.* Hemoglobin Level, Chronic Kidney Disease, and the Risks of Death and Hospitalization in Adults With Chronic Heart Failure. *Circulation* 2006;**113**:2713-2723. doi: 10.1161/circulationaha.105.577577

7. Gurwitz JH, Magid DJ, Smith DH*, et al.* Contemporary Prevalence and Correlates of Incident Heart Failure with Preserved Ejection Fraction. *The American Journal of Medicine* 2013;**126**:393-400. doi: 10.1016/j.amjmed.2012.10.022

8. McKee PA, Castelli WP, McNamara PM, Kannel WB. The Natural History of Congestive Heart Failure: The Framingham Study. *New England Journal of Medicine* 1971;**285**:1441-1446. doi: 10.1056/nejm197112232852601

9. Chen W, Yao J, Liang Z*, et al.* Temporal Trends in Mortality Rates among Kaiser Permanente Southern California Health Plan Enrollees, 2001-2016. *Perm J* 2019;**23**. doi: 10.7812/TPP/18-213

10. de Groot V, Beckerman H, Lankhorst GJ, Bouter LM. How to measure comorbidity. a critical review of available methods. *J Clin Epidemiol* 2003;**56**:221-229. doi: 10.1016/s0895-4356(02)00585-1

11. Charlson ME, Pompei P, Ales KL, MacKenzie CR. A new method of classifying prognostic comorbidity in longitudinal studies: development and validation. *J Chronic Dis* 1987;**40**:373-383. doi: 10.1016/0021-9681(87)90171-8

12. Coleman KJ, Ngor E, Reynolds K*, et al.* Initial validation of an exercise "vital sign" in electronic medical records. *Med Sci Sports Exerc* 2012;**44**:2071-2076. doi: 10.1249/MSS.0b013e3182630ec1

13. Kunisaki KM. CPAP adherence and cardiovascular disease: beware of the healthy adherer effect. *Sleep Breath* 2020;**24**:599-600. doi: 10.1007/s11325-019-01849-9
