# Supplementary material for: Positive airway pressure therapy and cardiovascular events in obstructive sleep apnoea: an observational clinical cohort study
Source: Sleep Med. Author manuscript; Available in PMC 2026 May 26. (PMC13203992; doi:10.1016/j.sleep.2025.108732)
Supplement: Multimedia component 3 [file NIHMS2166968-supplement-Multimedia_component_3.docx]

STROBE Statement—checklist of items that should be included in reports of observational studies

|  | | | Item No. | Recommendation | Page  No. | | | Relevant text from manuscript | |
| --- | --- | --- | --- | --- | --- | --- | --- | --- | --- |
| **Title and abstract** | | | 1 | (*a*) Indicate the study’s design with a commonly used term in the title or the abstract | 1 | | | Line 2 | |
|  |  |  |  | (*b*) Provide in the abstract an informative and balanced summary of what was done and what was found | 2  3 | | | Lines 1-22  Lines 1-3 | |
| Introduction | | | | | | | |  | |
| Background/rationale | | | 2 | Explain the scientific background and rationale for the investigation being reported | 4  5 | | | Lines 1-23  Lines 1-14 | |
| Objectives | | | 3 | State specific objectives, including any prespecified hypotheses | 5 | | | Lines 11-14 | |
| Methods | | | | | | | |  | |
| Study design | | | 4 | Present key elements of study design early in the paper | 5 | | | Lines 11-14, 18-19 | |
| Setting | | | 5 | Describe the setting, locations, and relevant dates, including periods of recruitment, exposure, follow-up, and data collection | 5  6 | | | Lines 18-23  Lines 1-6  Supplemental material | |
| Participants | | | 6 | (*a*) *Cohort study*—Give the eligibility criteria, and the sources and methods of selection of participants. Describe methods of follow-up  *Case-control study*—Give the eligibility criteria, and the sources and methods of case ascertainment and control selection. Give the rationale for the choice of cases and controls  *Cross-sectional study*—Give the eligibility criteria, and the sources and methods of selection of participants | 5  6 | | | Lines 19-23  Lines 1-7  Supplemental material | |
|  |  |  |  | (*b*) *Cohort study*—For matched studies, give matching criteria and number of exposed and unexposed  *Case-control study*—For matched studies, give matching criteria and the number of controls per case | N/A | | | N/A | |
| Variables | | | 7 | Clearly define all outcomes, exposures, predictors, potential confounders, and effect modifiers. Give diagnostic criteria, if applicable | 6  7 | | | Lines 9-23  Lines 1-15  Supplemental material | |
| Data sources/ measurement | | | 8* | For each variable of interest, give sources of data and details of methods of assessment (measurement). Describe comparability of assessment methods if there is more than one group | Supplement | | | Supplemental material | |
| Bias | | | 9 | Describe any efforts to address potential sources of bias | 6 | | | Lines 2-7 | |
| Study size | | | 10 | Explain how the study size was arrived at | 8 | | | Line 18  Supplemental material | |
| Quantitative variables | | 11 | | Explain how quantitative variables were handled in the analyses. If applicable, describe which groupings were chosen and why | 6  8 | | | | Lines 16-22  Line 5  Supplemental material |
| Statistical methods | | 12 | | (*a*) Describe all statistical methods, including those used to control for confounding | 7 | | | | Lines 16-23  Supplemental material |
|  |  |  |  | (*b*) Describe any methods used to examine subgroups and interactions |  | | | | Supplemental material |
|  |  |  |  | (*c*) Explain how missing data were addressed |  | | | | Supplemental material |
|  |  |  |  | (*d*) *Cohort study*—If applicable, explain how loss to follow-up was addressed  *Case-control study*—If applicable, explain how matching of cases and controls was addressed  *Cross-sectional study*—If applicable, describe analytical methods taking account of sampling strategy | 6 | | | | Lines 2-3 |
|  |  |  |  | (*e*) Describe any sensitivity analyses | N/A | | | | N/A |
| Results | | | | | | | | | |
| Participants | | 13* | | (a) Report numbers of individuals at each stage of study—eg numbers potentially eligible, examined for eligibility, confirmed eligible, included in the study, completing follow-up, and analysed | Figure  8 | | | | Figure 1  Lines 17-21 |
|  |  |  |  | (b) Give reasons for non-participation at each stage | Figure | | | | Figure 1 |
|  |  |  |  | (c) Consider use of a flow diagram | Figure | | | | Figure 1 |
| Descriptive data | | 14* | | (a) Give characteristics of study participants (eg demographic, clinical, social) and information on exposures and potential confounders | Tables  9 | | | | Tables 1 and 2  Lines 1-10 |
|  |  |  |  | (b) Indicate number of participants with missing data for each variable of interest | N/A | | | | N/A |
|  |  |  |  | (c) *Cohort study*—Summarise follow-up time (eg, average and total amount) | Tables | | | | Tables 1 and 2 |
| Outcome data | | 15* | | *Cohort study*—Report numbers of outcome events or summary measures over time | Tables  9 | | | | Table 3  Lines 13-21 |
|  |  |  |  | *Case-control study—*Report numbers in each exposure category, or summary measures of exposure | N/A | | | | N/A |
|  |  |  |  | *Cross-sectional study—*Report numbers of outcome events or summary measures | N/A | | | | N/A |
| Main results | | 16 | | (*a*) Give unadjusted estimates and, if applicable, confounder-adjusted estimates and their precision (eg, 95% confidence interval). Make clear which confounders were adjusted for and why they were included | Tables  9  10  11 | | | | Table 3  Lines 22-23  Lines 1-23  Lines 1-11 |
|  |  |  |  | (*b*) Report category boundaries when continuous variables were categorized | 6  8 | | | | Lines 16-22  Line 5  Supplemental material |
|  |  |  |  | (*c*) If relevant, consider translating estimates of relative risk into absolute risk for a meaningful time period | N/A | | N/A | | |
| Other analyses | 17 | | Report other analyses done—eg analyses of subgroups and interactions, and sensitivity analyses | | N/A | N/A | | | |
| Discussion | | | | | | | | | |
| Key results | 18 | | Summarise key results with reference to study objectives | | 11 | Lines 14-23 | | | |
| Limitations | 19 | | Discuss limitations of the study, taking into account sources of potential bias or imprecision. Discuss both direction and magnitude of any potential bias | | 15  16  17 | Lines 19-23  Lines 1-23  Lines 1-9 | | | |
| Interpretation | 20 | | Give a cautious overall interpretation of results considering objectives, limitations, multiplicity of analyses, results from similar studies, and other relevant evidence | | 11  12  13  14  15  16  17 | Lines 14-23  Lines 1-23  Lines 1-23  Lines 1-23  Lines 1-23  Lines 1-23  Lines 1-9 | | | |
| Generalisability | 21 | | Discuss the generalisability (external validity) of the study results | | 13  14 | Lines 18-23  Lines 1-2 | | | |
| Other information | | |  | | | | | | |
| Funding | 22 | | Give the source of funding and the role of the funders for the present study and, if applicable, for the original study on which the present article is based | | 18 | Lines 1-4 | | | |
